# Indents

**Published:** 1914-02-15

**Authors:** 


### INDENTS.

☞Brief Articles of Technical or Scientific Value will receive notice and comment. Contributions invited.

*Danger of Breaking Down too Fast the Granulation Tissues Around Infected Teeth.*

An important thought in the operative treatment of pyorrhea is one which has been impressed upon the author by repeated misfortune, viz, that it is unwise to disturb more than two or three teeth at a sitting, on account of the fact that the wall of granulation tissue which hedges in pyorrheal infections is broken down through the effort to free the root surface of its infection. When the wall of granulation is thus broken down, large numbers of bacteria are introduced directly into the circulation. If only two or three teeth are disturbed, the individual can absorb and destroy the dose. If, however, through the anxiety of the patient to have his work rapidly completed, a large area involving six or eight teeth is disturbed, the patient is liable to have a pneumonia supervene, or an active joint infection, or an acute nephritis, or he may have a sharp chill and considerable temperature for two or three days. Having noticed examples of each of these four types of post-operative disturbance described, the author limits the area treated in any one mouth to two or three teeth at one sitting.

In line with the thought just discussed is another bearing on the same question. The writer also forbears attempting to stamp out a mouth infection immediately preceding a grave surgical operation in other parts of the body. He prefers to have the mouth operated on from ten days to two weeks before subsequent surgical interference is necessary; for the patient is more liable to harm on account of the breaking-down of the wall of granulation tissue, which has existed for perhaps years, and which is the one factor which has prevented a pneumonia, a joint infection, or a septicemia, any one of which serious disturbances may follow after this protective wall of leucocytes is broken down. During the skinning of the root surface the patient may have absorbed a sufficient amount of bacteria to so weaken his resistance as to react very poorly under the added stress of a grave surgical operation; whereas, if sufficient length of time elapses for healing to have occurred before subsequent operation takes place, the patient is in a very much better condition to resist a serious operation in other parts of the body. The importance of splinting teeth which have lost so much bone that there is not enough left to hold them rigidly after infection is stamped out should be considered.—Dr. Hartzell, in Nov. *Cosmos.*

*Right Amount of Rubber in Flasks.* If rubber is measured before packing there will be no doubt about results. Warm the flask slowly to heat bearable to the hand; separate, remove wax; measure in water and replace with same amount of rubber adding a trifle for any loss of wax.

Heat flask and absorb any wax; pack; put in clamp and vulcanize in steam, as then the model is always dry and hard. I do not think a model need be wet at any stage.

This does away with opening flask to examine before vulcanizing or having a lot of rubber squeeze out and you will always know just how the plate is coming out.—H. R. Staley, in *Review.*

*Bromural.* Dr. T. Ulkan, of Berlin, reports that Bromural-Knoll is excellent for nervous and frightened children who resist all attempts at dental treatment. This preparation seems to exert a selective action upon the cerebrum without giving rise to disagreeable after-effects.

Doses of 5 grains, or 1 tablet, quiet the children after about 20 minutes. Their resisting powers are gone, they seem apathetic and allow themselves to be treated readily. Several cavities can be filled in succession without trouble, and injections and extractions can be easily done. If larger operations are necessary, such as chiselling or manipulations on the mucous membranes, 10 grains, or 2 tablets, Bromural may be given to children over 10 years old. After 20 minutes the injection can be made, and 10 minutes later the operation may be successfully performed. In this way three roots have been resected at one sitting for a very frightened and excited child without any struggling on the part of the patient.—*Deutsche Zahnaerztliche Wochenschrift*, Nov. 15, 1913.

*A New Protective Feature.* The following is an extract from the amendment to the By-Laws adopted by the Massachusetts State Dental Society last year, which I recommend for your consideration:

"Active members of the society, in good standing, shall be entitled, in accordance with conditions specified in the following sections of this article, to receive, without personal expense therefor, advice, and court service of an attorney in the employ of the society, for the purpose of conducting their defense in any court in this commonwealth, when they are unjustly accused of malpractice.

"Active members of this society desiring to avail themselves of the privileges provided in this article shall make application therefor, in writing, to the society's law committee, and shall show to its satisfaction that they are members in good standing in the society, with all obligations to the society fully discharged. They shall also furnish the law committee, at its request, a complete and accurate statement of their connection with the treatment of persons upon which complaints against them are based, giving dates of treatment, names and addresses of persons cognizant of facts and circumstances necessary to a clear and definite understanding of all matters in question, and shall furnish such other relevant information, if possible, as may be required of them by the law committee or the attorney of the society."—Dr. R. J. Wenker, in *Review*.

*Setting Up Full Dentures Without Use of Articulator.* This only applies to a case where one denture is being constructed and the teeth are being occluded to the remaining teeth or denture in the opposing jaw. Use rigid base plate and build wax to contour desired. Insert in mouth and test bite. When trial-plate strikes uniformly start arranging teeth. Cut sufficient wax on left of median line to place the two incisors and the cuspid. When arranged properly remove trial-plate and seal teeth with hot wax being careful not to heat remaining wax or trial-plate. Chill trial-plate; cut sufficient wax away and place trial-plate in mouth; arrange bicuspids to occlude with opposing teeth; remove, seal teeth and chill. Trim wax to allow molars to be placed; arrange molars in mouth and repeat preceding technique. While the teeth are being arranged on the one side the wax of trial-plate is maintaining the proper relation on the opposite side. Having arranged all the teeth on the one side proceed with same technique on the opposite side.—Geo. P. Brenner, in *Review.*

*Hemophilia Treated by Calcium Loctate.* In the October, 1913, New Jersey Dental Journal, Dr. C. F. Jones describes a serious case of Hemophilia successfully treated by giving Calcium Lactate every hour, giving twenty grains for the first dose and gradually reducing the dose to five grains and then increasing the dose gradually until twenty grains are again given,. This was kept up for two weeks, the patient meanwhile being kept on a liquid diet. On three occasions, at long intervals, tooth extraction was followed by alarming hemorrhage, that was eventually controlled by the administration of the Calcium Lactate. The patients father and grandfather both died from this cause. This case as reported enforces the caution that in these cases watchfulness and care must be continued long after the bleeding ceases. There may be no hemorrhage for days and by some slight exertion a more severe hemorrhage than the first one, will ensue. In the case above reported it was three weeks before the hemorrhage entirely ceased. Absolute quiet and rest on the part of the patient are essential. The administration of the Calcium Loctate for a time previous to an operation will prevent the hemorrhage.—Dr. W. H. Trueman, in *Dental Brief.*

*Salol in Dentifrices.* A case of Eczema of the lips is reported from the use of a tooth powder containing Soap, Salol, Chalk, and Pumice. The chemical reaction of the Salol probably increased by the irritation of the Pumice was the cause of the Eczema, which promptly subsided when the use of the powder was discontinued.—*The Dental Record.*

*Porcelain Inlay Matrix.* Carefully prepare the cavity in such a manner that the impression may be removed, after having been chilled, without pulling or distortion. Any impression material may be used, preferably base plate gutta-percha. The use of a thin matrix is advocated, especially in mesial or distal cavities. The impression is then mounted on a thin dab of plaster to facilitate handling and occasionally to help out a thin margin.

The die is then made of copper amalgam, copper cement or any material of sufficient edge strength.

This die is mounted on a small bed plate accompanying the mechanical swager. After burnishing the platinum matrix with the burnishers and the assistance of a little cotton, it is placed in the swager and compressed. This can be repeated up to the last baking or as long as the operator thinks necessary.

This method obviates the liability of the matrix to become distorted by handling as is very often the case where the matrix is burnished in the cavity. It also widens the scope of the porcelain inlay as many inlays can be made by this method where it would be impossible to burnish a matrix accurately in the mouth.—H. H. Hayes, in *Dental Review.*

*Dentists Neglectful of Aseptic Practice.* I have visited many dental offices where this criticism would not apply, and I have visited others where the need of it was so pronounced that it was difficult for me to understand why educated people would patronize such men. I am pleased, however, to make this comment, that many of these disease spreaders have either changed their methods, ceased to practice dentistry, or are doing a poor business.

It should be borne in mind, in this connection, that there are a great many valuable patients constantly on a secret search for good, clean methods of practice. I have been convinced of this fact by noting how carefully educated patients secretly watch our actions. In case you are called to the telephone, or to shake hands with a friend, or perhaps to receive a telegram they will secretly observe whether you wash your hands, or dip them into an antiseptic solution before returning to the mouth to resume your work. In case you drop an instrument, or a crown on the floor, or in case you stop to wipe perspiration from your face, or reach into your pocket for change, or stop to turn on the electric fan, and a thousand other things which will contaminate your hands, they are conscious of your subsequent actions from the stand-point of asepsis.

When you recall the fact that laboratory experiments have proven pyorrhea alveolaris to be a contagious disease, that the state board of health has estimated that there are 12,500 cases of tuberculosis in the state, that an authority has estimated that 40 per cent of the American people have either inherited or acquired syphilis, and that the mouth is subject to many other diseases, and is a carrier of many pyogenic micro-organisms to which we are exposed, certainly this must bring a vivid picture to your mind of the constant danger of your daily practice, not only to your other patients, but to your family and to your own person as well.—Dr. R. J. Wenker, in *Review*.

